# Improvement of efficiency of brown coal biosolubilization by novel recombinant *Fusarium oxysporum* laccase

**DOI:** 10.1186/s13568-018-0669-1

**Published:** 2018-08-22

**Authors:** Natalia Kwiatos, Marzena Jędrzejczak-Krzepkowska, Bartosz Strzelecki, Stanisław Bielecki

**Affiliations:** Institute of Technical Biochemistry, Faculty of Biotechnology and Food Sciences, Lodz University of Technology, Stefanowskiego 4/10, 90-924 Lodz, Poland

**Keywords:** Laccase, Brown coal, Biosolubilization, Lignite, Biodegradation, Laccase mediator system

## Abstract

Clean coal technologies (e.g. coal biosolubilization) are of essential value, especially in Europe, where coal is the national wealth and other energy sources like crude oil are not available. *Fusarium oxysporum* LOCK 1134, the strain isolated from brown coal, efficiently biosolubilizes lignite. The obtained liquefied products contain 50% less sulfur and over 99% less mercury than the crude coal. Moreover, the liquefied coal can be modified further by laccase. In this study *F. oxysporum* laccase was expressed in *Pichia pastoris* for the first time and was assessed as an additional agent for coal degradation. The novel laccase contributes to humic and fulvic acids release from liquefied coal due to introduction of oxygen into coal structure. The effect is increased when a natural redox mediator, sinapic acid, is present in the reaction mixture—up to 30% and 80% respectively. Humic acids obtained by biological process are environmentally friendly fertilizers that may have stimulating effects on crop growth.

**Electronic supplementary material:**

The online version of this article (10.1186/s13568-018-0669-1) contains supplementary material, which is available to authorized users.

## Introduction

Brown coal is an important energy source in Europe. However, it is discouraged to be traditionally burnt due to its low calorific value and harmful substances that are created upon combustion, such as nitrogen and sulfur oxides. Liquefaction of coal offers converting the coal into cleaner fuel or creating a base for production of complex aromatic compounds (Igbinigie et al. 2008; Sekhohola et al. 2013). Apart from expensive and energy-demanding chemical methods several biological methods of coal solubilization are under investigation. The idea is to apply microorganisms (bacteria or fungi) or solubilizing agents produced by them (alkali substances, biosurfactants, chelators, enzymes) to degrade the polymer (Fakoussa and Hofrichter 1999; Gokcay et al. 2001; Wang et al. 2016). *Trametes versicolor*, *Trichoderma reesei*, *Bacillus mycoides* and *Gordonia alkanivorans* are few from the list of brown coal biosolubilizing microorganisms.

The products of biosolubilization are a mixture of humic, fulvic acids and other organic compounds. The chemical structure of the products enables their usage as excellent sorbents, for example in biosorption of various pollutants, especially heavy metals. Moreover, humic and fulvic acids may be used as mediators for biogeochemical reactions as they may perform function of oxidants or reducers. Brown coal has been proposed before as fertilizer and a component in soil remediation process (Sekhohola et al. 2013). Furthermore, high content of organic substances suggests application of liquefied coal in production of methane. The process of biosolubilization may be an initial step for methanogenesis. Addition of laccase will lead to further degradation of lignite which would result in easier methane production (Haider et al. 2013; Stephen and Park 2015). Liquefied brown coal may also be used as a raw material for production of various chemicals—alcohols, aromatic compounds, fatty acids, etc. The desired substances could be obtained by further chemical or enzymatic process or by application of microorganisms with specific metabolic abilities.

*Fusarium oxysporum* is an ascomycete reported as an excellent microorganism for brown coal biosolubilisation (Strzelecki et al. 2015). *F. oxysporum* possesses three genes that may encode for extracellular laccases (Kwiatos et al. 2015). Laccases have been enlisted as potential solubilising agents and also lignin degrading enzymes (Sekhohola et al. 2014; Wang et al. 2015; Munk et al. 2015; Ortner et al. 2015; Hämäläinen et al. 2018). It catalyzes oxidation of phenolic substrates with water as only by-product. Moreover, laccases are able to oxidize non-phenolic compounds indirectly, with help of redox mediators. In laccase mediator system (LMS) the enzyme oxidizes low molecular weight phenolic compound—mediator—and a very reactive intermediate product is formed. Next, it leaves the laccase active site and oxidizes other substrates which could not enter the enzyme active site on their own. The mediators are either synthetic ones like 2,2′-azino-bis(3-ethylbenzothiazoline-6-sulfonic acid) diammonium salt **(**ABTS), 1-hydroxybenzotriazole (HBT) or natural ones—lignin derivatives, such as sinapic acid, 2,6-dimethoxyphenol or *p*-coumaric acid. Laccases are enzymes useful in various fields—food and textile industry, biomedical diagnostics, biofuel production, organic synthesis and bioremediation (Pollegioni et al. 2015; Kaczmarek et al. 2017; Hilgers et al. 2018). Moreover, laccases have also potential in lignin valorization (Wang et al. 2015; Coconi Linares et al. 2018; Ghatge et al. 2018). In the study, we report heterologous expression of a new laccase from *F.* *oxysporum* for the first time and its assessment as a brown coal biosolubilizing agent.

## Materials and methods

### Materials

All chemicals used in the study were analytical grade and bought from Sigma (Saint Louis, USA) or Chempur (Piekary Śląskie, Poland). The brown coal used in the study was obtained from Bełchatów Brown Coal Mine (Poland). Coal particles with a diameter of 1–2 mm were subjected to pretreatment with 8M HNO_3_ as described earlier (Romanowska et al. 2015) and then subjected to microbial solubilization.

The microorganism used in the study was *F. oxysporum* LOCK 1134 (Centre of Industrial Microorganisms Collection—LOCK, WDCM105), strain isolated from brown coal from Bełchatów Brown Coal Mine, currently in pure culture collection at the Institute of Technical Biochemistry, Lodz University of Technology. *Pichia pastoris* KM71H from Invitrogen (Carlsbad, USA) was used for protein expression and *Escherichia coli* top10f’ (Invitrogen, Carlsbad, USA) for standard cloning procedures. pJET1.2 plasmid (Thermo Fisher Scientific, Walthman, USA) was used for basic molecular manipulations and pPICZαA vector (Invitrogen, Carlsbad, USA) was used for enzyme expression.

## Methods

### Construction of expression vector

All sequences encoding for Gr2 laccase with native signal sequence and without introns found on Fusarium Comparative Project website were aligned using Clustal W program (Kwiatos et al. 2015). The consensus sequence was subjected to codon optimization for *P. pastoris* expression. The resulting sequence (GeneBank accession number MH351668) was synthesized by GeneArt (Thermo Fisher Scientific, Regensburg, Germany) with *Eco*721 restriction site of 5′ end of the sequence and *Not*I on 3′ end of the sequence. The amplified with high fidelity polymerase DNA sequence was cloned to pJET vector. Having multiplied the vector by standard *E. coli* procedures (Evans 1990) the sequence was cut out by *Eco*721 and *Not*I restriction enzymes and ligated with pPICZα A with T4 DNA Ligase (5 U/µL) (Thermo Fisher Scientfic). The resulting vector was cut with *Sac*I restriction enzyme (Thermo Fisher Scientific) and transformed to electrocompetent *P. pastoris* KM71H according to the Invitrogen Easy Select Pichia Expression protocol. Positive transformants were selected on YPD plates (2% peptone from casein, 1% yeast extract, 0.4% dextrose, 1.5% agar) with zeocine (100 µg/mL). Then, the clones were cultivated in 96-deep well plates according to the procedure explained elsewhere (Weis et al. 2004). The clone producing the highest laccase activity in culture supernatant was chosen for further cultivations.

### Production of recombinant *F.* *oxysporum* laccase in *P. pastoris*

The recombinant *P. pastoris* KM71H was cultivated in Sixfors fermenter (InFors-HT, Bottmingen, Switzerland) of maximal volume of 500 mL according to Pichia Fermentation Process Guidelines with minor modifications. The fermentation basal salt medium was supplemented with 0.2 mM CuSO4. The fermentation started with inoculation with 20 mL preculture of OD_600_ 3 (10% initial volume). For the first 24 h the cultivation was at glycerol batch phase, when the oxygen spike appeared the glycerol fed-batch phase (18 mL/h/L of 50% glycerol with 12 mL/L pTM1 salt) begun and last for 4 h. The induction of protein expression started with the methanol fed-batch phase. The methanol was added gradually from 0 to 3 mL/h/L for 4 h and then the feed was constant till the end of the fermentation. Sampled were taken for measurements of laccase activity, protein concentration and wet biomass. The cultivation was stopped after 167 h from inoculation, the biomass was separated from supernatant with centrifugation (15 min, 4 °C, 8000×*g*). The protein was concentrated to the volumetric activity of 70 U/L (laccase preparation) on 30 kDa Tangential Flow Filtration Membrane Cassettes (Sartorius).

### ABTS assay

Laccase activity was measured at 25 °C by monitoring oxidation of 1 mM ABTS at 420 nm (ε420 = 36,000 M^−1^ cm^−1^) in McIlvaine buffer at pH 3.5. One unit of enzyme activity was defined as the amount of enzyme required to obtain 1 µmol of product per minute. Each sample was measured in duplicate.

### Plate screening for brown coal biosolubilization potential

*Fusarium oxysporum* LOCK 1134 was cultivated for 7 days at 30 °C on solid modified Czapek-Dox medium: glucose (10.0 g/L), sodium glutamate (2.0 g/L), NaNO_3_ (3.0 g/L), K_2_PO_4_ (2.0 g/L), MgSO_4_ (0.5 g/L), KCl (0.5 g/L), agar (15 g/L). The pH of the medium was set to 7 with 1M HCl before autoclaving. Small parts of brown coal pretreated with HNO_3_ were situated on fungi mycelium and incubated for 2 days at 30 °C.

### Brown coal biosolubilization by *F. oxysporum* LOCK 1134

*Fusarium oxysporum* LOCK 1134 was cultivated for 7 days at 30 °C on solid modified Czapek-Dox medium. The fungi were transferred from Petri dish to 500 mL flat-bottomed flasks with 100 mL liquid modified Czapek-Dox with 0.1% sterile coal powder as an inducer*. F.* *oxysporum* was cultivated for 3 days at 30 °C and 180 rpm. Next, 10 mL of obtained pre-culture was transferred to 100 mL of fresh medium. The fungus was cultivated in the same conditions for next 3 days. 5 g (5% w/v) of sterile pretreated coal was added to the prepared 3 days old culture. The experiment was conducted for 14 days at 30 °C and 180 rpm. Sterile medium with coal subjected to the same conditions was considered as a control. Having finished the incubation the biomass was centrifuged at 7740×*g* for 15 min and the obtained supernatant (liquefied coal) was further analyzed.

### Coal analysis

The fraction of solid coal and liquefied coal, were subjected to analysis. In order to assess the amount of produced humic and fulvic acids, the absorbance at 450 nm and 650 nm were measured accordingly with T80 + UV/VIS PG Instruments Ltd spectrophotometer. The amount of mercury in coal was determined by atomic absorption spectroscopy CV AAS and other elements were measured with EuroVector 3018.

### Liquified coal treatment with recombinant laccase

The liquefied coal was subjected to further degradation by recombinant enzyme preparation. 0.5 mL of solubilized coal (containing 15.2 mg/mL dry coal) was mixed with 0.5 mL of laccase preparation (0.035 U) with or without mediators and placed in 2 mL eppendorf tubes. The tubes were put on a rack and incubated in a shaking incubator (100 rpm, 40 °C) for 24 h. The following variants were assessed:

A. 0.5 mL of liquefied coal (pH 4.2) and 0.5 mL of laccase preparation
B. 0.5 mL of liquefied coal (pH 3) and 0.5 mL of laccase preparation
C. 0.5 mL of liquefied coal (pH 4.2), 0.5 mL of laccase preparation and 1 mM ABTS
D. 0.5 mL of liquefied coal (pH 4.2), 0.5 mL of laccase preparation and 5 mM ABTS
E. 0.5 mL of liquefied coal (pH 4.2), 0.5 mL of laccase preparation and 1 mM sinapic acid (SA)
F. 0.5 mL of liquefied coal (pH 4.2), 0.5 mL of laccase preparation and 5 mM sinapic acid.

All variants were prepared in triplicates. Every sample has its own control (instead of enzyme sterile water was added), also in triplicate.

### Analysis of the biodegradation process

Absorbance at 650 nm (fulvic acids) and 450 nm (humic acids) were measured after 1 h and 24 h by Multiskan™ GO Microplate Spectrophotometer (Thermo Scientific) in order to assess the amount of released acids. Fourier Transform Infrared Spectroscopy (FTIR) was used to determine the specific chemical groups of atoms in the structure of coal and its biosolubilization products. The FTIR analysis was performed using a Thermo Nicolet AVATAR 330 at the transmission range from 4400 to 400 cm^− 1^ after 24 h of incubation. Prior to FTIR analysis the samples were lyophilized.

## Results

### Expression and production of *F.* *oxysporum* laccase coding sequence in *P. pastoris*

**Fig. 1:**
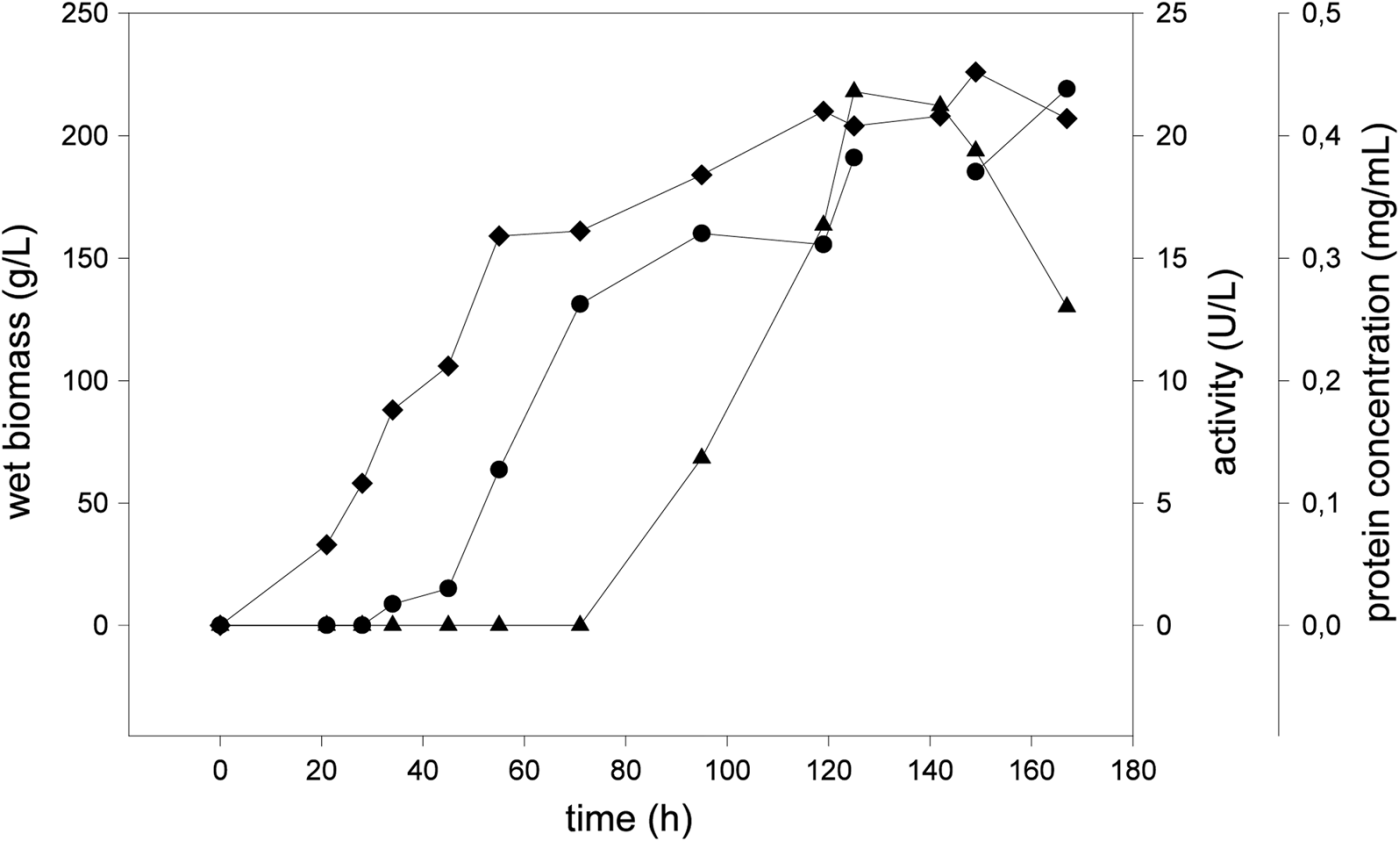
Dynamics of biosynthesis of recombinant *F.* *oxysporum* laccase in *P. pastoris* KM71H. Diamonds—wet biomass, triangles—laccase activity, circles—protein concentration

One of the potential *F.* *oxysporum* laccase coding sequences described before was synthesized with codon optimized for expression in *P.* *pastoris*. The 90 positive colonies that grew on YPD with zeocin were picked and cultivated in 96-deep well plate as explained previously (Weis et al. 2004). At the end of the cultivation the supernatant was harvested and subjected to ABTS assay to determine the laccase activity. The activities varied from well to well and the maximal reached 7 U/L while the average activity was 2.6 U/L. The best clone was picked and was produced by recombinant *P.* *pastoris* KM71H in 500 mL bioreactor according to the standard procedure. The samples were taken twice a day for wet biomass and laccase activity measurements. Laccase activity was detectable after 95 h from the inoculation and the fermentation was stopped when the activity in culture supernatant started to decrease. The maximum activity was 22 U/L reached in the culture stationary phase (Fig. 1). The laccase preparation was obtained by biomass separation and supernatant concentration.

### Plate screening for brown coal biosolubilization potential

**Fig. 2:**
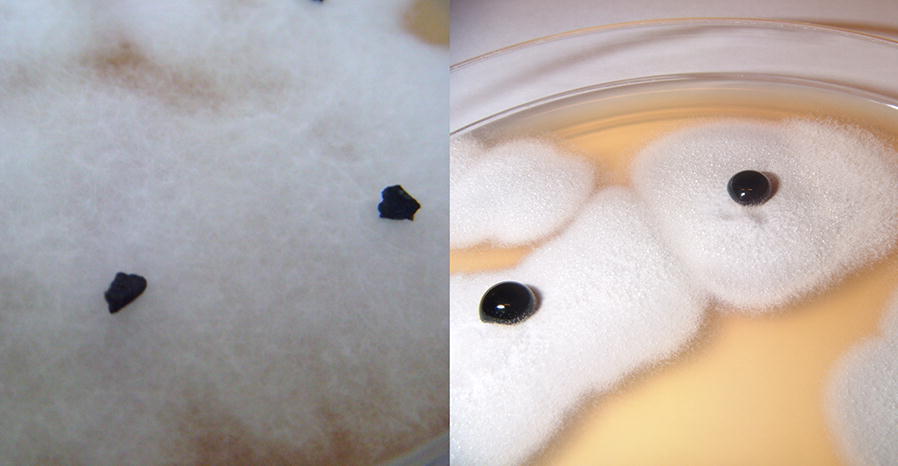
Brown coal biosolubilization by *F. oxysporum* LOCK 1134 on solid medium. On the left—crude brown coal on fungi mycelium, on the right—liquefied coal after 2 days at 30 °C

*Fusarium oxysporum* LOCK 1134 converts solid brown coal into dark liquid droplets after 2 days incubation at 30 °C. The experiment is presented in Fig. 2, in Additional file 1: Movie S1) a movie showing *F.* *oxysporum* LOCK 1134 solubilizing brown coal is presented.

### Brown coal biosolubilization by *F. oxysporum* LOCK 1134 and coal analysis

The brown coal was solubilized by *F.* *oxysporum* LOCK 1134 during the submerged cultivation of the fungi. Almost no humic and fulvic acids were formed in control sample (medium with coal), while the amount of humic acids formed by *F.* *oxysporum* solubilization reached 1474 mg/g in culture supernatant.

The elemental analysis revealed carbon content decrease in liquefied coal and increase in oxygen content (Table 1). Because of that we observe 50% decrease in calorific value of coal [calculated with Dulong equation (Yin et al. 2009)]. Coal biosolubilization led to significant decrease of sulfur and mercury content in liquefied products.

**Table 1:** Elemental analysis of crude coal, pretreated coal and liquefied coal

Sample/element	(%)						High heating values (MJ/kg)
	C	H	O	N	S	Other	
Coal	46.23 ± 0.11	5.38 ± 0.05	32.40 ± 0.02	0.30 ± 0.03	0.84 ± 0.05	14.8	18.0
Pretreated coal	44.0 ± 0.04	4.00 ± 0.13	30.00 ± 0.06	3.40 ± 0.06	0.4 ± 0.0	18.2	14.8
Liquefied coal	32.12 ± 0.06	4.06 ± 0.06	41.88 ± 0.04	5.79 ± 0.10	0.50 ± 0.04	15.6	9.2

### Liquid coal treatment with laccase preparation

#### Humic and fulvic acids release

**Fig. 3:**
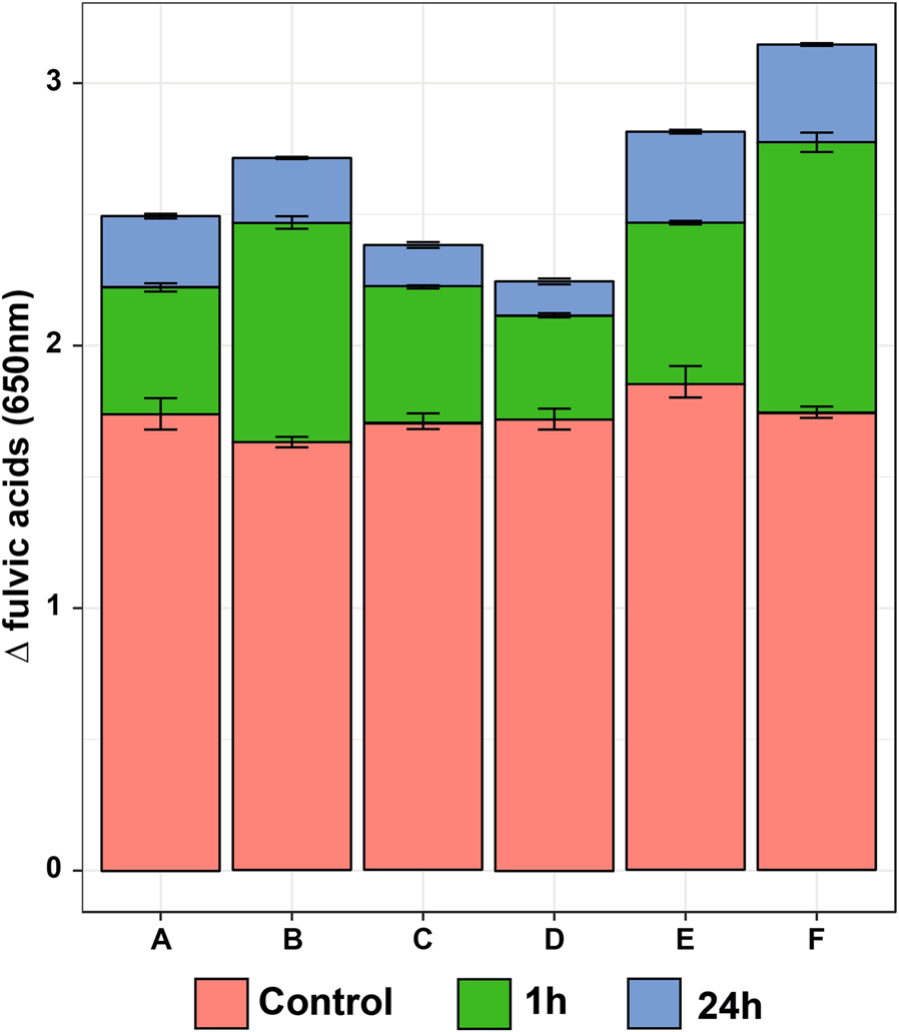
Increase of fulvic acids release during the enzymatic biodegradation of liquefied coal after 1 h and 24 h of the process. A. liquefied coal (pH 4.2) and laccase preparation, B. liquefied coal (pH 3) and laccase preparation, C. liquefied coal (pH 4.2), laccase preparation and 1 mM ABTS, D. liquefied coal (pH 4.2), laccase preparation and 5 mM ABTS, E. liquefied coal (pH 4.2), laccase preparation and 1 mM sinapic acid (SA), F. liquefied coal (pH 4.2), laccase preparation and 5 mM sinapic acid. Each variant had its own control—instead of enzyme sterile water was added

**Fig. 4:**
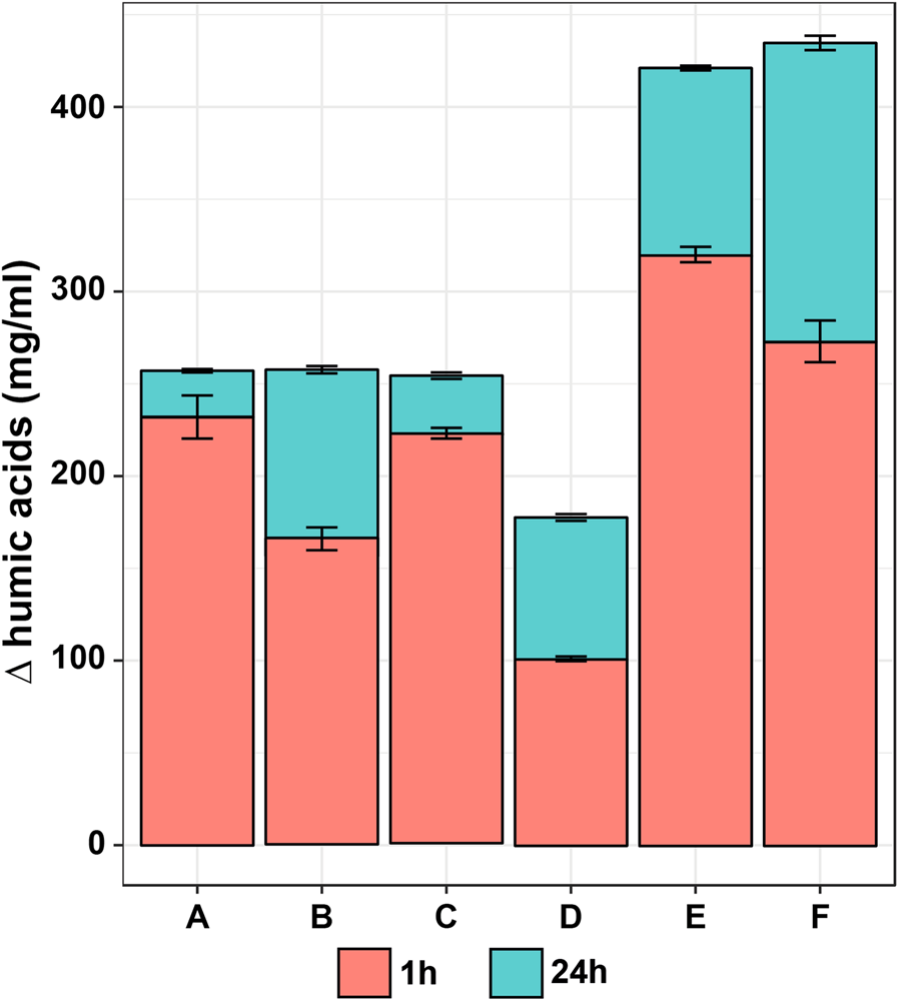
Amount of released humic acids during the enzymatic biodegradation of liquefied coal after 1 h and 24 h of the process. A. liquefied coal (pH 4.2) and laccase preparation, B. liquefied coal (pH 3) and laccase preparation, C. liquefied coal (pH 4.2), laccase preparation and 1 mM ABTS, D. liquefied coal (pH 4.2), laccase preparation and 5 mM ABTS, E. liquefied coal (pH 4.2), laccase preparation and 1 mM sinapic acid (SA), F. liquefied coal (pH 4.2), laccase preparation and 5 mM sinapic acid. Each variant had its own control—instead of enzyme sterile water was added. Control values were subjected from sample values

The biotransformation of liquid coal by recombinant laccase was done with and without presence of redox mediators. Humic and fulvic acids are released from lignite during the process for all six variants, which indicates that laccase took effective role in coal degradation. The most significant changes occur with presence of natural laccase mediator—sinapic acid, when its concentration is high (5 mM—variant F). On the other hand, the presence of ABTS does not benefit the reaction (variant C and D) (Figs. 3, 4).

#### FTIR analysis

**Fig. 5:**
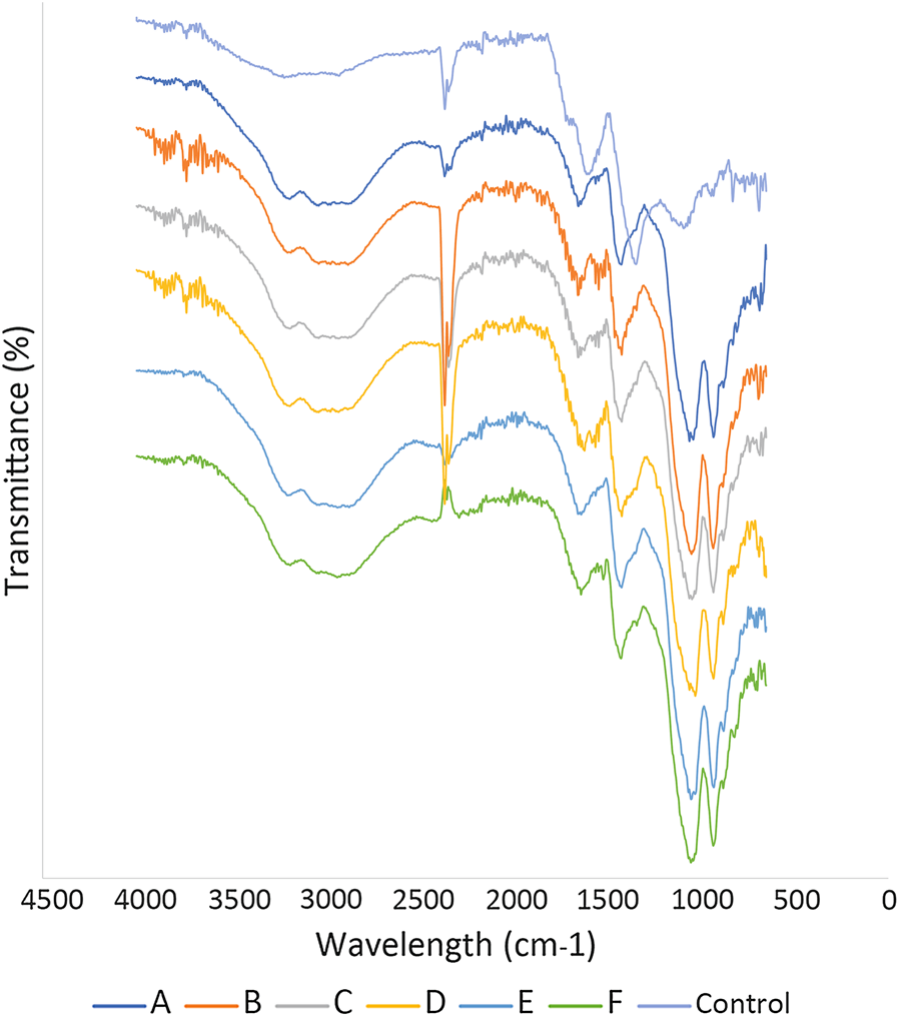
FTIR spectra for liquefied products. A. liquefied coal (pH 4.2) and laccase preparation, B. liquefied coal (pH 3) and laccase preparation, C. liquefied coal (pH 4.2), laccase preparation and 1 mM ABTS, D. liquefied coal (pH 4.2), laccase preparation and 5 mM ABTS, E. liquefied coal (pH 4.2), laccase preparation and 1 mM sinapic acid (SA), F. liquefied coal (pH 4.2), laccase preparation and 5 mM sinapic acid, control is liquefied coal with water

The release of humic and fulvic acids for all 6 variants is caused by similar events (Fig. 5). The lack of picks at 900 cm^−1^ and lower wavenumbers for all samples but control suggests the degradation of aromatic compounds. Moreover, higher transmittance at 1058 cm^−1^ signifies formation of ether bonds, while high transmittance at 1343 cm^−1^ formation of C–O bonds (in carboxylic groups), probably due to introduction of oxygen into coal structure. Other proves of oxidation of lignite by laccase is visible at wavenumbers around 1600—the increase of picks in this area suggests formation of esters, carboxylic groups or lactons. The differences in picks around 2360 cm^−1^ comes from various distribution of C=N bonds.

## Discussion

*Fusarium oxysporum* LOCK 1134 is a strain effectively solubilizing brown coal (Fig. 2). The amount of humic acids formed by *F.* *oxysporum* solubilization reached 1474 mg/g in culture supernatant. To compare, in compost average amount of humic substances, which are responsible for soil enrichment, reaches only 182 mg/g (Gusiatin and Kulikowska 2012). Recently, lignite derived humic acids were reported as an excellent fertilizer, that increase vegetable and wheat yield and soil quality index (Ahmad et al. 2015; Ciarkowska et al. 2017). Moreover, our results also supports these studies, showing that liquefied coal stimulates germination of seed and growth of plant roots (data not published yet).

The elemental analysis suggests that *F.* *oxysporum* metabolizes the carbon from coal—the amount of carbon decreases from 44 to 32%. The solubilization of lignite implies introduction of oxygen into coal structure to loosen the hard structure and make it more susceptible to other chemical changes. The biosolubilization by *F.* *oxysporum* LOCK 1134 ends with 10% increase in oxygen content in lignite (Table 1).

The maximal calorific value of coal (18 MJ/kg) and solubilization products (9.2 MJ/kg) was calculated with Dulong equation (Yin et al. 2009) (Table 1). The calorific value of coal is within the range of cited values (Höök et al. 2010). The biosolubilization of coal leads to 50% decrease in calorific value; mainly due to decrease in carbon and oxygen content. However, the product of coal solubilization could find an alternative of its application in energy production. Co-fermentation is applied for improvement of methane fermentation by dilution of the main substrate with addition of another organic matter. It is designed in the way that a proper carbon:nitrogen:phosphorous ratio is preserved (Sosnowski et al. 2008).

Biosolubilization aims to reduce impurities in coal, clear out lignite from mercury and sulfur or nitrogen that may form harmful oxides during the combustion of the material. Biosolubilization goes together with biodesulfurization—*F.* *oxysporum* LOCK 1134 is able to reduce sulfur content in almost 50% (Table 1). Comparing the results with previously published studies of biological biosolubilization with bacteria indicates that *F.* *oxysporum* more effectively dispose sulfur than *Gordonia alkanivorans* S7 and *Bacillus mycoides* NS1020 (Romanowska et al. 2015). S7 leaves 80% and NS1020 73% of sulfur content after solubilization in liquefied product, while *F.* *oxysporum* just 59%. Another benefit from biological liquefaction by *F.* *oxysporum* is reduction of mercury content in coal—it decreases from 164.62 µg/kg in pretreated coal to only 0.59 µg/kg in liquefied product. It is estimated that 1900 ton of mercury was emitted to the environment in 2010 all over the world by combustion of fossil fuels (Jozwik et al. 2014). Our results indicates that properly designed biosolubilization process has a great impact on reduction of harmfulness of exhausted gases.

The fungi possesses in its genome genes that encode laccases, however till now it was not known if extracellular laccases have an impact on lignite biosolubilization. The conducted research used *F.* *oxysporum* recombinant laccase expressed in *P.* *pastoris* to visualize the effect of the enzyme on coal structure. The study confirmed the ability of the laccase to improve the biodegradation process. The most optimum action of the enzyme is obtained if laccase-mediator system is in use and natural lignin mediators seem to be a better choice than synthetic ones. Nevertheless, many researches prove the benefit of enzyme engineering towards its specificity and optimal conditions to profit the processes conducted by them (Macellaro et al. 2014; Scheiblbrandner et al. 2017).

It is widely discussed if laccase can catalyze bond cleavage in lignin by itself (Munk et al. 2015; Tonin et al. 2016; Hilgers et al. 2018). Most scientists conclude that it is not possible due to the size of substrate binding pocket. The lignin either cannot enter the active site or cannot leave it inhibiting the laccase catalytic action. However, as the results suggests, the recombinant laccase from *F.* *oxysporum* is able to introduce changes in liquid coal structure without the addition of a mediator (40% less fluvic acids than with SA) (Fig. 3). The liquid coal is firstly partially degraded by fungus due to action of various agents, such as biosurfactants, alkali substances, chelators and enzymes. It is possible that in the liquid coal there are already smaller lignin parts that are little enough to act as mediators. They may be oxidized by laccase and then proceed and oxidize bigger molecules further. Another hypothesis is that while lignin is activated laccase leads to cleavage of the weakest bonds, for example the ether bonds (Munk et al. 2015).

For the changes in lignin structure to occur, one electron should be removed from lignin molecule. Then the surface becomes more reactive, which will lead to various changes: bond cleavage and depolymerization, change in functional groups (acetylation or demethylation) or coupling. Low molecular weight compounds like mediators may attach to lignin by radical coupling. The event may succeed in two ways: either in spreading of the mediator on the surface (grafting) or in continuation of radical polymerization which ends with lignin polymerization (Barneto et al. 2012; Munk et al. 2015). Grafting may promote solubilization of lignin, which is of interest for clean coal technologies techniques. The mechanism of polymerization may explain the formation of higher molecular weight compounds—humic acids (Figs. 3 and 4). Fulvic acids are formed during all the 6 processes; the highest amount is released when high concentration of sinapic acid is present. However, sole laccase also leads to formation of considerable amount of higher weight compounds—polymerization abilities of laccase are commonly acknowledged (Mikolasch and Schauer 2009; Munk et al. 2015; Pollegioni et al. 2015).

The recombinant laccase has its optimum at pH 3.5 and 40 °C. The experiment was conducted at 40 °C and pH 4.2—the pH of liquefied coal, except for variant B—the pH of liquefied coal was adjusted to pH 3 by 1M HCl. The release of humic acids are almost identical for variant A (laccase without mediators, pH 4.2) and variant B (laccase without mediators, pH 3), while the amount of produced fulvic acids are 13% higher for variant B (Fig. 3). This suggests that if the reaction took place for the variant with sinapic acid (E, F), probably the results would be even better. As it would not have industrial sense to change the pH of coal, the solution would be to engineer the enzyme to shift its pH optimum towards pH 4.2. The research on laccase directed evolution towards changes of its optimum reaction conditions have been done successfully in the past (Pardo et al. 2012; Mate et al. 2013).

The second bottleneck of the application that could be overcome by protein engineering is the stability of the enzyme in the most optimum conditions. As Fig. 3 presents the difference between the amount of fulvic acids released after 1 h of the experiment and 24 h is around 10% for all cases. Probably the enzyme is deactivated by too high temperature and too low pH after couple of hours; however the mediators may act even longer.

Heterologous expression of fungal laccases is a complex topic that brings many challenges. It is often necessary to optimize the cultivation conditions in order to obtain detectable level of laccase expression. The duration, oxidation of culture, pH of medium, concentration of copper ions and ethanol that could enhance the permeability of membrane and enhance secretion are only few to mention in long process of expression optimization. It is often required to enhance the secretion and expression levels by protein engineering. For example *Pycnoporus cinnabarinus* laccase was heterologously expressed in *Saccharomyces cerevisiae* at level of 0.003 U/L and then due to medium optimization and cost-intensive directed evolution enhanced 8000 fold to 24 U/L (Camarero et al. 2012). *Trametes versicolor* LCC1 was expressed in *P. pastoris* and its activity in 7-day culture supernatant was 2 U/L, when it reached 173 U/L after the optimization of culture conditions (Colao et al. 2006). *F. oxysporum* laccase was expressed in *P. pastoris* with maximal level of 7 U/L in 96 deep-well format, while cultivated in bioreactor, the expression of laccase was increased over threefold (Fig. 1).

Although, it is still unknown in which extend the extracellular laccases help the fungi in biosolubilization process, the addition of adequately tailored biocatalyst may be of interest for many industrial applications. Clean coal technologies are of essential value, especially in Europe, were coal is the national wealth and other energy sources like crude oil are not available.

## Additional file

**Additional file 1: Movie S1.** This video shows a time lapse movie of the solubilization of Polish brown coal (lignite) by *Fusarium oxysporum* LOCK 1134. Brown coal pieces were placed on the mycelium of a fungus and incubated for several days at room temperature. The video was made by automatically taking a picture of the petri dish every 10 minutes. Author: Manu de Groeve.

## Abbreviations

ABTS: 2,2′-azino-bis(3-ethylbenzothiazoline-6-sulfonic acid) diammonium salt
SA: sinapic acids
